# Successful glycemic control using a flash glucose monitoring system for a pregnant woman with diabetes: a case report

**DOI:** 10.1186/s40200-017-0327-1

**Published:** 2017-11-06

**Authors:** Miyako Kishimoto, Saori Tamada, Yoko Oshiba

**Affiliations:** 1 Clinical Research Center, Department of Medicine, International University of Health and Welfare, Tokyo, Japan; 2 Department of Internal Medicine, Sanno Hospital, 8-10-16 Akasaka Minato, Tokyo, 107-0052 Japan; 3 Department of Obstetrics and Gynecology, Sanno Hospital, 8-10-16 Akasaka Minato, Tokyo, 107-0052 Japan

**Keywords:** Gestational diabetes mellitus, Impaired glucose tolerance, Flash glucose monitoring system, Insulin

## Abstract

**Background:**

Glucose control for pregnant women with glucose intolerance is important, as hyperglycemia may adversely affect the mother and the fetus.

**Case presentation:**

We report the case of a pregnant Japanese woman who experienced gestational diabetes mellitus during her first pregnancy and developed impaired glucose tolerance after the delivery. During her second pregnancy with twins, she required up to 75 units of injected insulin to control her postprandial hyperglycemia and occasionally experienced hypoglycemia. We used a newly developed flash glucose monitoring system, which allowed her to successfully achieve ideal glycemic control and experience an uncomplicated delivery.

**Conclusion:**

We suggest that this flash glucose monitoring system may be clinically effective for similar cases that involve pregnant women with abnormal glucose tolerance.

## Background

Gestational diabetes mellitus (GDM) is glucose intolerance during pregnancy, and is associated with both maternal and fetal risks [1–5]. Women with GDM have an increased risk of developing impaired glucose tolerance (IGT) or type 2 diabetes [6–8]. We recently encountered a pregnant Japanese woman who was diagnosed with GDM during her first pregnancy and subsequently developed IGT after the delivery. During her second pregnancy, she required large doses of injected insulin and faced the possibility of premature labor. However, intensive care from an interdisciplinary healthcare team allowed her to achieve a successful delivery without serious adverse outcomes.

Appropriate glycemic control in this case was achieved using a novel sensor-based flash glucose monitoring (FGM) system (FreeStyle Libre™; Abbott Diabetes Care, Alameda, CA) that does not require routine finger pricks, which eliminates pain and inconvenience from the testing process [9]. Data regarding the measured interstitial glucose levels are transferred to a display, which also shows a glucose trend arrow and a graph of the glucose readings from the previous 8 h. These data can be uploaded and printed by using FreeStyle Libre software. Compared to conventional self-monitoring of blood glucose (SMBG) using capillary strips, the FGM system reduces the incidence of hypoglycemia among patients with type 1 diabetes, without a deterioration in their glycated hemoglobin (HbA1c) levels [10]. The FGM system can also be used as part of intensive insulin therapy for type 2 diabetes, which reduces the incidence of hypoglycemia without altering the patient’s HbA1c levels [11]. According to the manufacturer’s instruction, sensor glucose readings may not be accurate, and a finger prick test using a glucometer is required when 1) glucose levels change rapidly, as interstitial fluid glucose levels may not accurately reflect blood glucose levels, 2) the displayed value is not in accordance with the symptoms, and 3) hypoglycemia needs to be confirmed [12–14].

Therefore, the FGM system cannot be used as an alternative to conventional SMBG. However, this novel system appears to be a safe and effective adjunctive tool and our findings highlight its utility during the treatment of pregnant women with glucose intolerance.

## Case presentation

A 30-year-old pregnant Japanese woman was diagnosed with GDM at 23 weeks and 3 days of gestation during her first pregnancy, based on the results of a 75-g oral glucose tolerance test (OGTT). Her blood glucose levels before the test, after 60 min, and after 120 min were 81 mg/dL, 151 mg/dL, and 180 mg/dL, respectively. She was not obese before the pregnancy, had a height of 163 cm, a body weight of 54 kg, a body mass index (BMI) of 20.3 kg/m^2^ and had no family history of diabetes. She received dietary counselling, began SMBG (ONETOUCH UltraVue™ Johnson & Johnson, New Brunswick, NJ), and successfully achieved good glycemic control until the day of her delivery (a healthy boy with a birth weight of 3164 g). At 3 months after the delivery, she completed a follow-up 75-g OGTT, and the blood glucose levels before the test, after 30 min, after 60 min, and after 120 min were 82 mg/dL, 146 mg/dL, 102 mg/dL, and 189 mg/dL, respectively. Her plasma insulin levels before the test and after 30 min were 2.20 μU/mL and 33.2 μU/ml, respectively. Therefore, she was diagnosed with IGT based on these results, an insulinogenic index of 0.48, and a homeostatic model assessment of insulin resistance (HOMA-IR) result of 0.44. Thereafter, her HbA1c level was regularly monitored and ranged from 5.4% to 5.7%.

**Fig. 1:**
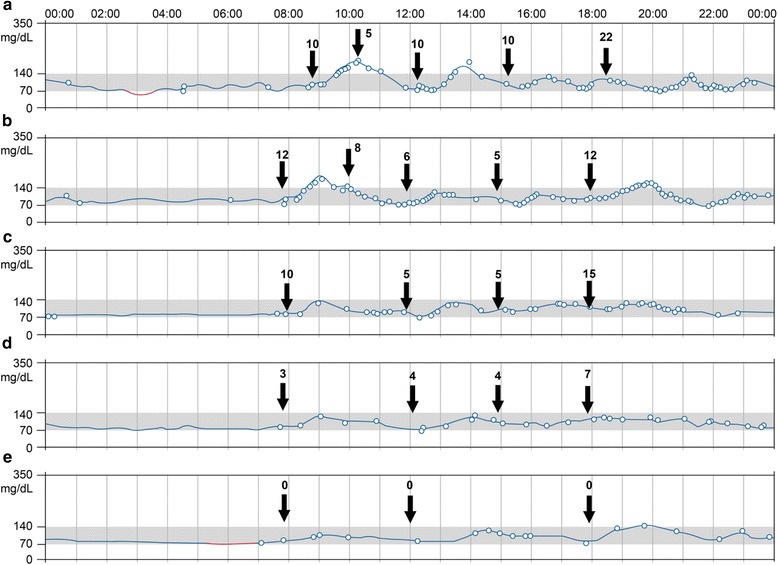
The results of continuous glucose monitoring using the sensor-based flash glucose monitoring system. The arrows indicate the timing of the patient’s meals and insulin injections. The numbers above the arrows indicate the numbers of the units for each insulin injection. The patient needed 57 units of injected insulin on day 8 (2200 kcal in 5 meals) (**a**), 43 units of injected insulin on day 10 (1960 kcal in 5 meals) (**b**), 35 units of injected insulin on day 17 (1960 kcal in 4 meals) (**c**), 18 units injected insulin on day 38 (1960 kcal in 4 meals) (**d**), and no injected insulin at 3 days after the delivery (2200 kcal in 3 meals) (**e**)

At the age of 33 years, the patient became pregnant with twins. At approximately 5 weeks of gestation, her SMBG began showing high postprandial glucose levels (up to 140–180 mg/dL), and she began self-administered insulin injections using insulin aspart (Novo Nordisk) three times per day before each meal. The doses of these insulin injections increased with gestational age, and reached 20–22 units before each meal. At 31 weeks and 3 days of gestation, the patient was urgently admitted to our hospital due to premature labor contractions. We initiated a continuous intravenous infusion (500 mL/day of 5% glucose solution containing 50 mg of ritodrine) that was continued until the day of her delivery. The patient experienced adverse effects from the ritodrine, required complete bed rest, and her glucose levels kept increasing (2200 kcal/day in 3 meals). Even with 25 units of injected insulin before each meal, her postprandial glucose levels increased to 211 mg/dL at 2 h after lunch, and she occasionally experienced hypoglycemia (59 mg/dL at 3 h after lunch or 50 mg/dL at approximately 10 PM). Thus, to reduce the fluctuations in her glucose levels, her food intake (2200 kcal/day with 59% carbohydrates, 15% protein, and 26% fat) was separated into 5 meals: first breakfast at 8 AM, second breakfast at 10 AM, first lunch at noon, second lunch at 3 PM, and dinner at 6 PM. The patient also received 1 cup of yoghurt immediately before going to sleep (Fig. 1a). However, the patient subsequently experienced abdominal distension and difficulty eating, and we reduced her intake to 1960 kcal/day (Fig. 1b).

Based on the increased meal frequency, the patient required more frequent SMBG and insulin injections, which she found to be painful and depressing. Thus, we introduced the FGM system, which does not require finger punctures, to reduce her physical and emotional burden. During the first few days after its introduction, the FGM system exhibited discrepancies with the results from the conventional SMBG method, especially when her glucose levels were <70 mg/dL or >180 mg/dL. Therefore, she measured her glucose levels using both methods when her glucose levels were abnormally high or low. The patient also experienced difficulty consuming the second breakfast because of the short interval between the first and second breakfasts. Thus, her intake was revised to 1960 kcal/day (53% carbohydrates, 21% protein, and 26% fat) in 4 meals: breakfast at 8 AM, first lunch at noon, second lunch at 3 PM, and dinner at 6 PM. The re-distributed meals and flash glucose monitoring system allowed the patient to achieve good glycemic control, and the doses of the injected insulin decreased to 35 units (Fig. 1c) and then to 18 units (Fig. 1d). Her HbA1c level was maintained, ranging from 5.1% to 5.2% (these values were considered as a reference due to her anemia), and her glycoalbumin levels ranged from 10.6% to 11.6% during pregnancy. She gained 10.5 kg during her pregnancy and subsequently delivered twins via Caesarean procedure (2280 g and 2778 g) at 37 weeks and 1 day of gestation, without any adverse events or hypoglycemia. After the delivery, the patient stopped all insulin injections and had glucose levels of 70–140 mg/dL throughout the day with a normal puerperium diet (2200 kcal/day in 3 meals) (Fig. 1e). The patient and her twins were discharged in healthy states at 7 days after the delivery.

## Discussion

Recently, results of studies on the use of the FGM system in terms of accuracy and patient’s satisfaction were favorable [10, 11, 13–16], and the availability of the FGM system as an alternative to glucose monitoring can be positively evaluated [13]. However, this system has certain precautions and disadvantages, including the mismatch between sensor glucose reading and SMBG due to the time lag of glucose from the intravascular to interstitial compartment [17, 18], and this effect is particularly pronounced when glucose levels are rapidly increasing or decreasing [14]. In addition, the use of glucose sensor is less accurate during hypoglycemia [16]. As for the precautions and limitations stated in the manufacturer’s manual, the FGM system should not be used by pregnant women or individuals on dialysis because the system has not been evaluated in these populations [12]. During pregnancy, changes in the water content in the body compartments may affect the accuracy of glucose measurements when the FGM system is used. Therefore, the evaluation of the patient’s sensor reading required special precation, and a simultaneous SMBG must also be performed. Thus, the patient used the FGM system and conventional SMBG for the first week, and we monitored the differences between the two results. We observed several discrepancies during periods of hypoglycemia (e.g., conventional SMBG: 86 mg/dL, the FGM system: 68 mg/dL) and hyperglycemia (conventional SMBG: 218 mg/dL, the FGM system: 182 mg/dL). However, the two results were frequently similar, and any differences were within the allowable range. Therefore, the patient mainly used the FGM system and only occasionally used conventional SMBG from the second week after the introduction of the FGM system to the day of her discharge.

Blood glucose monitoring at 4–7 times per day can help improve perinatal and pregnancy outcomes [19–21], and our patient originally measured her glucose levels four times per day (before breakfast and after each meal), based on the recommendations for patients with GDM and pre-existing diabetes [3]. However, after her diet was changed to 4–5 meals, she had to perform conventional SMBG at least 8 times per day, based on the recommendations of the American College of Obstetricians and Gynecologists [22] and the American Diabetes Association. For women with type 1 or type 2 diabetes and GDM, these recommendations target fasting glucose levels of ≤95 mg/dL (5.3 mmol/L) and either 1-h postprandial glucose levels of ≤140 mg/dL (7.8 mmol/L) or 2-h postprandial glucose levels of ≤120 mg/dL (6.7 mmol/L) [3]. Thus, she would have required more frequent SMBG to adjust her insulin injections, and the increased frequency of these tests caused her to become stressed and depressed.

The FGM system does not require painful finger pricks, and patients are willing to perform 3 times more frequent glucose monitoring compared to conventional SMBG [10]. After introducing this novel system, our patient completed more frequent glucose monitoring, and the improved blood glucose monitoring allowed her to select more appropriate injected insulin doses. This improved accuracy resulted in a decrease in her daily dosage from 75 units to 18 units. Furthermore, the patient was able to detect and manage early-stage hypoglycemia before it progressed any further.

Even before the introduction of ritodrine at her hospitalization, the patient required up to 66 units of injected insulin per day (20–22 units before each meal). Information regarding the effects of hyperglycemia during twin pregnancies is sparse, and it remains unclear whether twin pregnancies are associated with increased risks of maternal, fetal, and neonatal complications, compared to singleton pregnancies [23–29]. Nevertheless, we suggest that the large required doses of injected insulin can be partially explained by her twin pregnancy.

## Conclusion

In conclusion, we suggest that the FGM system is safe and effective for glycemic management of pregnant women with abnormal glucose tolerance, and especially for women who require frequent SMBG because of their frequent meals and insulin injections. This new system could be a less invasive alternative for patients who need careful and frequent glucose monitoring and could be a beneficial system for people who care for these patients. A large study of pregnant women with glucose intolerance is needed to identify patients who will experience the greatest benefits from this novel monitoring system.

## Abbreviations

FGM: Flash glucose monitoring
GDM: Gestational diabetes mellitus
IGT: Impaired glucose tolerance
OGTT: Oral glucose tolerance test
SMBG: Self-monitoring of blood glucose
